# Microalgae, old sustainable food and fashion nutraceuticals

**DOI:** 10.1111/1751-7915.12800

**Published:** 2017-08-15

**Authors:** José L. García, Marta de Vicente, Beatriz Galán

**Affiliations:** ^1^ Department of Environmental Biology Centro de Investigaciones Biológicas (CIB) (CSIC) Madrid Spain; ^2^ Department of Applied Biotechnology Institute for Integrative Systems Biology (I2SysBio) (Universidad de Valencia‐CSIC) Valencia Spain

## Abstract

Microalgae have been used for centuries to provide nourishment to humans and animals, only very recently they have become much more widely cultured and harvested at large industrial scale. This paper reviews the potential health benefits and nutrition provided by microalgae whose benefits are contributing to expand their market. We also point out several key challenges that remain to be addressed in this field.

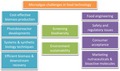

## Algae against malnutrition

Achieving an adequate nutrition is a growing global concern with the increase in world population. Therefore, cost‐effective sources of nutrients that can easy and rapidly produce large amounts of products of high nutritional value are needed. Algae can provide a significant source of a diverse number of essential nutrients to support human health. Algae are ubiquitous throughout the world and have persisted and thrived in numerous types of environments. Algae are a diverse group of photosynthetic organisms both unicellular (microalgae) and multicellular (macroalgae) that have the ability to grow rapidly, efficiently use light energy, fix atmospheric CO_2_, and produce more biomass per hectare than vascular plants.

This review assesses the properties of microalgae that are microscopic photosynthetic organisms present in both marine and freshwater environments. The term microalgae, in applied phycology, usually includes the microscopic algae *sensu stricto* and the photosynthetic bacteria (i.e. cyanobacteria), formerly known as Cyanophyceae. The cell structure is eukaryotic in microalgae and prokaryotic in cyanobacteria, but in terms of biomass, they are both considered as a potential source of energy, fuel, food and other many interesting commercial products. The most abundant microalgal classes are Cyanophyceae (blue‐green algae), Chlorophyceae (green algae), Bacillariophyceae (including the diatoms) and Chrysophyceae (including golden algae). Currently, many microalgae are acquiring increasing biotechnological interest because they can produce a number of nutraceutical compounds. These molecules can be defined as nutrients from food products that not only supplement the diet but also facilitate the prevention or treatment of a disease and/or disorder. Recent articles review the state of the art on the biotechnological production and use of microalgae (Chew *et al*., 2017; Odjadjare *et al*., 2017), but specially as food and feed (Yaakob *et al*., 2014; Liu and Chen, 2016; Bleakley and Hayes, 2017), and nutraceuticals (Nicoletti, 2016; Yan *et al*., 2016; Bilal *et al*., 2017; Wells *et al*., 2017). The aim of this article was to provide an overview of the current challenges on the use of microalgae as food, feed and nutraceutical products.

## Old foods and new medicines

Microalgae have been used as a human food source or nutritional supplements for hundreds of years. Aztecs used the cyanobacterium Spirulina (*Arthrospira platensis, Arthrospira maxima*) from Lake Texcoco (Mexico) circa AD 1300. Spanish chroniclers described local fishermen collecting blue‐green masses from the lakes that were prepared as a dry cake, known as ‘tecuitlatl’. For centuries, the population in Chad has been harvesting Spirulina (known as ‘dihé’) from Lake Kossorom at the north‐east fringe of Lake Chad and using it for food on a daily basis. *Nostoc,* filamentous cyanobacteria, has been also widely used as food. The species *N. commune*,* N. flagelliforme* and *N. punctiforme* are traditionally consumed in China, Mongolia, Tartaria and South America (known as ‘fa cai’ and ‘lakeplum’). In Japan, another edible cyanobacterium *Aphanotheca sacrum* (formerly *Phylloderma sacrum*) is considered a special delicacy known as ‘suizenji‐nori’. The filamentous green algae *Spirogyra* and *Oedogonium* are also used as a dietary component in Burma, Thailand, Vietnam and India. Despite this background, the modern era of microalgal and cyanobacterial biotechnological developments began in the early 1940s and gained momentum with the first Algal Mass‐Culture Symposium that was held at Stanford University in 1952. Since then, numerous technologies and approaches for mass controlled cultivation of microalgae (e.g. open ponds, specialized photobioreactors) have been suggested for their commercial exploitation apart from being harvested from natural environments. Moreover, Spirulina was declared by the United Nations World Food Conference of 1974 as the best food for the future, and United Nations World Health Organization (WHO) stated that Spirulina represents an interesting food for multiple reasons, for example, it is rich in iron and protein and is able to be administered to children without any risk (Geneva, Switzerland June 8Th, 1993). During the sixtieth session of the United Nations General Assembly (Second Committee, Agenda item 52), IIMSAM (International institution for the use of microalgae Spirulina against malnutrition) initiated a revised draft resolution on the ‘Use of Spirulina to combat hunger and malnutrition and help achieve sustainable development’. As a follow‐up on this resolution, the United Nations Food and Agriculture Organization (FAO) prepared a draft position on Spirulina that was presented in 2008 (FAO, 2011). Thus, microalgae can be an alternative to achieve food of high quality with a low environmental impact, because they can be cultivated in non‐cultivable lands.

Nowadays, *Chlorella* and Spirulina are widely commercialized in health food stores gaining worldwide popularity because they are one of the most nutritious foods known to man. These microalgae are also used to feed many types of animals (e.g. cats, dogs, aquarium fish, ornamental birds, horses, poultry, cows and breeding bulls). Moreover, other microalgae such as *Tetraselmis, Isochrysis, Pavlova, Phaeodactylum, Chaetoceros, Nannochloropsis, Skeletonema* and *Thalassiosira* are also used as feeds in aquaculture (Gouveia *et al*., 2008). However, microalgae are also potential sources of valuable bioactive compounds with a wide application as nutraceuticals. The nutraceuticals that can be extracted from microalgae are very abundant and diverse (Table 1) and have been proposed to treat a large number of diseases; nevertheless, some of these properties must be still confirmed by solid clinical trials.

**Table 1:** Microalgae products

Products	Microalgae	References
*Food*	*Chlorella,* Spirulina, *Odontella auriata*,* Tetraselmis chuii Aphanizomenon flosaquae, Nostoc, A. sacrum, Spirogyra, Oedogonium*	Gantar and Svirčev (2008), Liu and Chen (2016) and Bleakley and Hayes (2017)
*Feed*	*Chlorella,* Spirulina, *Tetraselmis, Isochrysis, Pavlova, Phaeodactylum, Chaetoceros, Nannochloropsis, Skeletonema, Thalassiosira*	Gouveia *et al*. (2008) and Yaakob *et al*. (2014)
*Vitamins & carotenoids*		
Vitamin B12	*Chlorella,* Spirulina	Begum *et al*. (2016), Henríquez *et al*. (2016) and Sun *et al*. (2016)
Vitamin E	*Porphyridum cruentum*	
β‐carotene	*D. salina, Haematococcus pluvialis, Synechococcus, Nanocloropsis gaditana*	
α‐carotene	*Chlorella*	
Astaxanthin	*D. salina, H. pluvialis, Chlorella*	
Lutein	*Murellopsis sp., Chlorella, Scenedesmus almeriensis, Auxenochlorella protothecoides*	
Zeaxanthin	*D. salina, Chlorella, Synechococcus, N. gaditana*	
Canthaxanthin	*Scenedesmus komarekii, D. salina, Chlorella*	
Fucoxanthin	*Phaeodactyllum tricornutum*	
Phytoene	*D. salina*	
Phytofluene	*D. salina*	
Violaxanthin	*Chlorella, Synechococcus, N. gaditana*	
Antheraxanthin	*Chlorella*	
Echinenone	*Botrycoccus braunii*	
Cryptoxanthin	*D. salina*	
*Phycobiliproteins*		
Phycocyanin	Spirulina	Sonani *et al*. (2016)
Phycoerythrin	*Porphyridium*	
Allophycocyanin	Spirulina	
*Chlorophyll*		
Chlorophyll A	*A. flosaquae*	Bishop and Zubeck (2012)
*PUFAs*		
Eicosapentaenoic acid (EPA)	*Phaeodactylum tricornutum, Monodus subterraneus, P. cruentum, Chaetoceros calcitrans, Nannochloropsis, Schizochytrium*	Pereira *et al*. (2012) and Adarme‐Vega *et al*. (2014)
Docosahexaenoic acid (DHA)	*Crypthecodinium cohnii, Isochrysis galbana, Pavlova salina, Schizochytrium*	
Linoleic acid	*D. salina*	
γ‐linolenic acid	Spirulina	
Oleic acid	*D. salina,* Spirulina	
Lauric acid	Spirulina	
*Polysaccharides*		
Sulphated polysaccharides	*Porphiridium spp*.	Delattre *et al*. (2016) and Xiao and Zheng (2016)
Nostoflan	*Nostoc flagelliforme*	
*Sterols*		
Brassicasterol, sitosterol, and stigmasterol	*D. salina, Dunaliella tertiolecta*	Luo *et al*. (2015)
*Phenolic & volatile compounds*		
Phenolic compounds	*Chlorella, Nostoc, Anabaena, Tolypothrix, Chlamydomonas*	Plaza *et al*. (2010) and de Morais *et al*. (2015)
β‐Cyclocitral	*D. salina*	
α‐ and β‐Ionone	*D. salina*	
Neophytadiene	*D. salina*	
Phytol	*D. salina*	
Pentadecane	*Synechocystis sp*.	
Heptadecane	Spirulina	
*Extracts*		
Total	*Chlorella, Chlamydomonas*	Nakashima *et al*. (2009)
Lipids	*Nostoc, Ulkenia*	
Carotenoids	*Chlorella*	

Carotenoids are in general richly coloured molecules consisting of a class of more than 600 naturally occurring organic pigments synthesized by plants, algae and bacteria which play different physiological roles and because of that they offer huge nutraceutical values. β‐Carotene is used as a food colouring as well as to improve the health and fertility of grain‐fed cattle. Astaxanthin is well known to have powerful antioxidant and anti‐inflammatory activities capable of preventing or reducing protein degradation, macular degeneration, rheumatoid arthritis, cardiovascular diseases, neurodegenerative diseases, such as Parkinson's, and cancers. Lutein, zeaxanthin and β‐carotene prevent premenopausal breast cancer, while cryptoxanthin and α‐carotene prevent cervical cancer. Moreover, fucoxanthin has antiobesity and anticancer activities. Chlorophyll is a photosynthetic pigment found in all photoautotrophic organisms and has been shown to stimulate liver function recovery and increase bile secretion (Bishop and Zubeck, 2012). It also repairs cells, increases haemoglobin in blood and promotes rapid cell growth. Chlorophyll is reported to have antimutagenic, anticarcinogenic and antioxidant activities. Traditionally, is used in the food industry as a colorant due to the increasing consumer demands for natural foods.

Phycobiliproteins are hydrophilic proteins bonded to the photosynthetic pigments phycobilins, mainly found in cyanobacteria and some red algae (Sonani *et al*., 2016). Nowadays, one of the main uses of Spirulina is for the extraction of phycocyanin that is exploited for many purposes (e.g. natural dyes, fluorescent agents/markers, cosmetics) but as nutraceutical it has been commercialized as antioxidant, anti‐inflammatory, neuroprotective or hepatoprotective agent.

PUFAs (polyunsaturated fatty acids) contain three or more double bonds on a fatty acid skeletal chain containing 18 or more carbon atoms. In particular, *n*‐3 PUFAs have been shown to be effective in preventing or treating several diseases (e.g. cardiovascular disorders, cancer, type 2 diabetes, inflammatory bowel disorders, asthma, arthritis, kidney and skin disorders, depression and schizophrenia).These compounds are now mainly derived from fish oil but due to several concerns (e.g. overexploitation of marine reserves, accumulation of toxic compounds in fishes, peculiar smell, unpleasant taste, oxidative instability), there is an increasing interest in using microalgae as potential alternative source of PUFAs.

Many microalgae are producers of structurally diverse exopolysaccharides (EPS) that are widely used in the food industry as thickeners and gelling additives (Liu *et al*., 2016). EPS have been shown to have multiple pharmaceutical activities (e.g. antioxidant, antitumor, antihyperlipidemia, antibacterial and anticoagulant activities).

Other bioactive compounds of interest that can be obtained from microalgae are sterols (Luo *et al*., 2015). This family of compounds has become well known for their ability to reduce LDL cholesterol and promote cardiovascular health. Moreover, sterols have been reported to be involved in anti‐inflammatory and antiatherogenicity, anticancer and antioxidative activities and can provide protection against nervous system disorders, such as autoimmune encephalomyelitis, amyotrophic lateral sclerosis or Alzheimer's disease.

Several microalgae have total phenolic contents that are similar to or even higher than several popular fruits and vegetables (de Morais *et al*., 2015). Their biological functions are very diverse highlighting antioxidant, anti‐inflammatory, antimicrobial activities and slow the progression of certain cancers and reduce the risks of cardiovascular disease, neurodegenerative diseases and diabetes.

Finally, microalgae have been reported to be a source of volatile compounds having antimicrobial activity (Plaza *et al*., 2010).

## Concerns associated with consumption of microalgae

Although the nutritional value of microalgae has been well documented, their digestibility and overall nutritional value depend not only on the genetic traits of individual strains but also on the technological processes used for biomass production. Some studies mention the taste, texture, colour and odour of microalgal biomass as potential bottlenecks, while other indicate that microalgae have the desired taste, texture and odour. These properties are therefore relevant issues to be considered in the development of food products or ingredients based on microalgae.

One of the limiting factors of using large quantities of microalgae for human consumption is the high content of nucleic acids that are metabolized to uric acid and might result in adverse health effects, such as gout or kidney stones (Gantar and Svirčev, 2008).

Cyanobacterial biomass, besides being produced in controlled conditions, is also harvested for commercial purposes from natural environments (e.g. Klamath Falls Lake (USA) and Lake Chad (Chad)) and although natural habitats are advantageous with respect to using nutrient sources naturally present in those lakes, the quality of this biomass is less consistent compared to that produced in controlled systems and requires frequent and extensive monitoring.

Some cyanobacteria can produce potent hepatotoxins and neurotoxins, and thus, when microalgae are grown in open basins or harvested from natural lakes, there is a risk of biomass becoming contaminated with alien toxic cyanobacteria and other biological and non‐biological contaminants (Grobbelaar, 2003).

## Engineering microalgae

Several microalgae (e.g. *Synechococcus*,* Synechocystis*,* Anabaena*,* Chlamydomonas reinhardtii*,* Nanocloropsis gaditana, Ostreococcus tauri*,* Porphyridium tricornutum)* are currently established for genetic engineering approaches (Doron *et al*., 2016). Curiously, Spirulina has proved to be extremely recalcitrant to transformation. Genetic modification of eukaryotic microalgae and cyanobacteria is now mainly studied in the laboratory because for industrial production, the process should be scaled up and applied outdoors. Under contained conditions genetically modified organisms (GMOs) can be cultured and processed in such a way that protective measures can be used to prevent their direct contact with the environment; however, for industrial production outdoors, it will be impossible to work under such conditions and the current legislations hinder the implementation of these processes. In this sense, CRISPR/Cas9 technology has been recently used to efficiently generate stable targeted gene mutations in microalgae, using *P. tricornutum* (Nymark *et al*., 2016) and *Nannocloropsis* as model organisms (Wang *et al*., 2016). Whether organisms modified using editing technologies will be considered as GMOs is now under discussion in many developed countries.

The metabolic optimization of microalgae as biocatalysts requires a systems level understanding of their metabolic and physiological processes and genome‐scale metabolic network reconstructions may be essential in achieving these goals and several genome‐scale metabolic models of microalgae are already available (Gudmundsson and Nogales, 2015).

Although metabolic engineering and synthetic biology of microalgae are promising approaches to increasing the production of nutraceuticals under contained conditions (Gimpel *et al*., 2015), there are still very few examples of advances in this matter, for example PUFAs (Hamilton *et al*., 2016) and carotenoids (Wang *et al*., 2014).

## Challenges for developing large‐scale photobioreactors

Natural waters (lakes, lagoons, ponds) or artificial ponds have been used in the past to grow microalgae. Open‐air systems depend on natural light for illumination, and although they are inexpensive to install and run, they suffer from many problems (e.g. cultures are not axenic; predators can damage the culture; weather variability hampers a proper control of the system). The species that are currently being cultured in open ponds are extremophiles capable of growing in a highly selective environment (high pH, salinity or temperature) to preclude the growth of contaminants (Xu *et al*., 2009).

The future of microalgal biotechnology depends on the development of large‐scale photobioreactors (PBRs) capable of operating under defined optimal conditions with minimal contamination risks (Wang *et al*., 2012). Compared with open‐air systems, closed systems can circumvent most of their problems, but a development of more economical, and efficient closed culturing systems are needed. Several types of closed PBRs have been designed so far including tubulars, plastic bags, flat plates, airlift and bubble columns, stirred tank reactors or even building‐window reactors (Xu *et al*., 2009; Enzing *et al*., 2014). In all cases, an efficient use of light is a major constraint and makes the scale‐up difficult. Thus, new developments in lighting and its control are needed to make closed culture systems competitive. In this sense, irradiance oriented, optical fibres or diodes providing internal illumination have been used to enhance light intensity (Glemser *et al*., 2016; Sun *et al*., 2016). Designing surfaces with proper materials, functional groups or surface coatings, to prevent microalgal adhesion is also essential for solving the typical biofouling problems of PBRs (Zeriouh *et al*., 2017).

On the other hand, scale‐up methodologies have to be improved for achieving efficient light provisions, minimal carbon dioxide losses, and efficient mixing and removal of generated oxygen (Gupta *et al*., 2015). Microalgal processes have similarities to traditional bioprocesses but demand unique monitoring requirements (Havlik *et al*., 2016). Modularization and computational fluid dynamics have been used to optimize the structural configurations of PBRs for scale‐up (Xu *et al*., 2009). Continuous culture cultivation and the development of PBRs for life support systems in the space add new challenges in this field.

Finally, in spite of the problems found to develop large‐scale closed systems, it is worth to mention that although closed reactors could be too small for some products like biofuels, they can be reasonable for high value‐added products like nutraceuticals. Moreover, contained PBRs allow the production of nutraceuticals by using recombinant microalgae.

## Safety and regulatory issues

As stated above, food safety is a relevant aspect to be considered in microalgae technology, especially for production in open‐air systems (Enzing *et al*., 2014). Thus, the regulations on the utilization and production of microalgal products are taken into account in developed countries by their regulatory bodies.

There are two EU regulations that most closely affect production and commercialization of microalgae‐based products for food and feed (Vigani *et al*., 2015), that is, the Regulation on Food Safety and the Regulation on Novel Food and Novel Food Ingredients. Regulation about GMOs for food and feed is excluded of the list because, at this moment, there are not recombinant microalgae at commercial phase. In 2002, the European Parliament and the Council adopted General Food Law Regulation (EC No 178/2002) laying down the general principles and requirements of food law. It sets out a framework for the development of food and feed legislation both at EU and national levels. It also sets up an independent agency responsible for scientific advice and support, the European Food Safety Authority (EFSA). Novel Food is defined as food that has not been consumed to a significant degree by humans in the EU prior to 1997, when the first Regulation on novel food came into force. Between 1997 and 2014, there have been around 170 applications for authorization across the EU (7–10 applications/year). A new Novel Food regulation for EU [Regulation (EU) 2015/2283, repealing Regulation (EC) No. 258/97], was adopted on November 2015 (coming into effect in January 2018) and aims to increase the efficiency of the authorization procedure, to enable a quicker delivery of safe, innovative food to market and to remove unnecessary barriers to trade, while ensuring a high level of food safety. In EU, several microalgae products have been already approved according to the regulations in force, like *O. auriata* (2002) and *T. chuii* (2014) approved as novel foods and the oils from *Schizochytrium* or *Ulkenia* and astaxanthin from *H. pluvialis,* approved as novel food ingredients.

In the United States, the FDA (Food and Drug Administration) regulates the safety of food including algal products. According to the classification of the Center for Food Safety and Applied Nutrition, algal biomass is classified as ‘other dietary supplement’. Spirulina, *Chlorella*,* Dunaliella*,* Haematococcus, Schizochytrium, P. cruentum* and *C. cohnii* are classified as food sources falling into the GRAS (Generally Recognized As Safe) category. Other products derived from microalgae classified by FDA as GRAS are oils obtained from *Schyzochitrium* and *Ulkenia*, as well as a whole microalgal protein powder and a lipid ingredient derived from *Chlorella*.

In addition to the EU and the USA, other developed countries have specific regulations. For instance, Food Standards Australia New Zealand (FSANZ) regulates the use of novel food ingredients, such as products from microalgae. According to the Food Standard Code, they have granted permission for using the dried marine microalga *Schizochytrium*, which is rich in omega‐3 long‐chain polyunsaturated fatty acid (DHA), as a novel food ingredient in a limited range of foods. Health Canada is advising consumers to apply caution when using non‐Spirulina cyanobacterial products. In addition, it does not allow therapeutic claims for substances sold as foods.

## Market trends

In the last years, there is a growing interest of consumers on ‘healthy food’ or products ‘good for you’. Healthy food comprises food products, ingredients or dietary supplements that may benefit your overall health or may prevent, treat or cure some diseases. Health‐conscious consumers are driving the demand for products that aim to promote better health, increase longevity and prevent the onset of chronic diseases (PriceWaterhouse&Coopers, 2009). Therefore, the use of microalgal biomass or its derived metabolites has become an innovative approach for the development of healthier food products (Gouveia *et al*., 2008).

Looking at the current microalgae‐based products volume in the market, dried whole algae Spirulina is the largest, and more than 12 000 tons of Spirulina biomass are produced every year (at about 30 US$/kg); nearly 70 per cent is produced in China, India and Taiwan. Worldwide, *Chlorella* producers cultivate an estimated 5000 tons per year. The market volume of other microalgae is followed by *D. salina* (about 3000 tn for carotene), *A. flosaquae* (about 1500 tn for food), *H. pluvialis* (about 700 tn for astaxanthin), *C. cohnii* (500 tn of DHA) and *Shizochytrium* (20 tn of DHA).

According to a recent market report published by Credence Research, the algae products market is expected to reach US$ 44.6 Bn by 2023, expanding at a CAGR (Compound Annual Growth Rate) of more than 5.2% from 2016 to 2023 (www.credenceresearch.com/report/algae-products-market). For example, the global Spirulina market is expected to register a CAGR of 10% during the forecast period and is estimated to be valued at nearly US$ 2000 Mn by 2026, owing to factors such as increased application of Spirulina in cosmetics or the recent approval of phycocyanin as natural blue colour for food.

As per Transparency Market Research, the global nutraceutical market (including functional food and beverages ingredients, dietary supplements, and personal care and pharmaceuticals), which was valued at US$ 182.60 Bn in 2015, will rise to US$ 278.96 Bn by 2021, exhibiting a CAGR of 7.3% for this period (www.transparencymarketresearch.com/pressrelease/global-nutraceuticals-product-market.htm). Based on revenues, functional food ingredients represented the largest product segment with a share of over 31% in 2014. As the functional food industry continues experienced relevant sales growth, global food firms remain focused on the development of new functional food and beverages with added ingredients (Bagchi and Nair, 2017). For instance, the *Chlorella* ingredients market is poised to register a CAGR of 25.4%, to reach US$ 700 Mn by 2022.

There are also promising market niches for specific nutraceuticals. Thus, the global market for carotenoids is projected to reach US$ 1.7 Bn by 2022 (www.strategyr.com/Marketresearch/Carotenoids_Market_Trends.asp). Global market for both synthetic and natural source astaxanthin in aqua feed, nutraceuticals, cosmetics, and food and beverages is projected to reach 670 tn valued at US$ 1.1 Bn by 2020 (www.researchandmarkets.com/reports/ 3129287/global‐astaxanthin‐market‐sources‐technologies). In addition, as mentioned above, microalgae can be an alternative source of PUFAs, which have been traditionally obtained from fish or extracted from fish oil. EPA/DHA ingredients market size from dietary supplements application could surpass 95 000 tn by 2022 (www.gminsights.com/industry-analysis/EPA-DHA-omega-3-ingredients-market).

## Conclusions and future prospects

**Figure 1:**
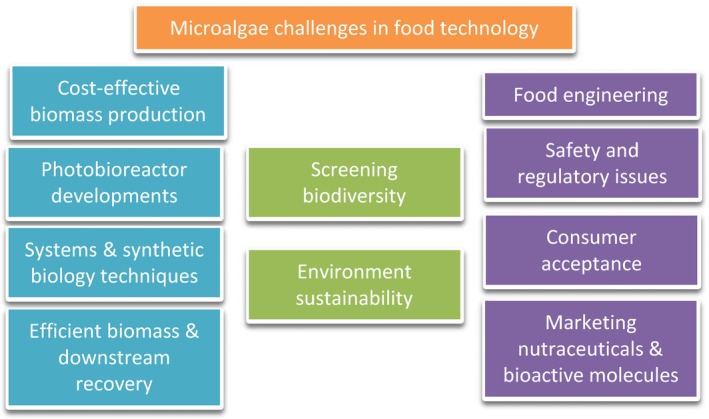
Key challenges for improving production and consume of microalgae as food, feed and nutraceuticals.

Although microalgae have been used for centuries to provide nourishment to humans and animals, only very recently they have become much more widely cultured and harvested at large industrial scale. Their roles in providing health benefits and nutrition as well as their applications in the energy, and cosmetic industries are contributing to expand their market but still several key challenges remain to be addressed in this field (Fig. 1).

The effects of microalgae and their nutraceutical derived products have been tested in many nutritional studies worldwide, but still many positive health benefits are likely to be discovered with their increased consume as food and feed supplements. Their potential for treating and preventing many types of diseases should improve the interest and promote research activities into their value mainly for human health. Thus, more intensive research on identifying new functional compounds from microalgae would give more health benefits to humans. As microalgae still remain as one of the most unexplored groups of organisms in the world, more research in bioprospecting is needed. For instance, microalgae can be utilized as an alternative and sustainable source of PUFAs; however, very few species are used for PUFAs’ production and more in‐depth investigation studies and continued isolation of new strains are required.

Key areas that require further attention on microalgal research are *omics* technologies and systems and synthetic biology (Reijnders *et al*., 2014; Gimpel *et al*., 2015; Gudmundsson and Nogales, 2015). Although more than 30 microalgal genomes have reportedly been sequenced (Guarnieri and Pienkos, 2015), new *omics* data are necessary to understand and characterize the pathways involved in the production of nutraceuticals and the regulatory mechanisms that trigger the production of these metabolites, such as nutrient deprivation, biotic and abiotic stress. Much work is still required on the genetic transformation and manipulation of microalgal strains to favour a high‐level production of target compounds.

The design and construction of large PBRs as well as the downstream processes for harvesting and dewatering the microalgal biomass and extraction and purification of target nutraceutical compounds represent the major costs of microalgae production plants. However, in recent years, due to high focus of producing high amounts of biofuels as renewable energy from microalgae, major improvements have been made in these biotechnological operations. Nevertheless, more research on the field of constructing PBRs or open pond systems made of inexpensive and environmentally friendly materials and development of less energy demanding and inexpensive downstream techniques is required to make profitable the production of natural compounds derived from microalgae versus synthetic ones.

## Conflict of Interest

None declared.
